# Aberrant long non-coding RNA cancer susceptibility 15 (CASC15) plays a diagnostic biomarker and regulates inflammatory reaction in neonatal sepsis

**DOI:** 10.1080/21655979.2021.1996514

**Published:** 2021-12-06

**Authors:** Jia Song, Ruihua Yu, Jianhong Qi, Xiaokang Wang, Qingqing Shen

**Affiliations:** Department of Neonatology, Shandong Provincial Hospital Affiliated to Shandong First Medical University, Shandong, 250021, China

**Keywords:** Neonatal sepsis, CASC15, miR-144-3p, inflammation

## Abstract

Neonatal sepsis (NS) is one of the important causes of neonatal death. There are many studies to confirm the role of long non-coding RNA (lncRNA) in neonatal infectious diseases. This study aimed to explore the level of cancer susceptibility 15 (CASC15) and its effect on inflammatory response in NS. Seventy-nine neonatal pneumonia (NP) patients and 80 NS patients were enrolled in this study. Reverse Transcription-quantitative PCR (RT-qPCR) was used to determine the expression levels of CASC15 and miR-144-3p. Receiver operating characteristic (ROC) curve was drawn to evaluate the diagnostic value of CASC15 in NS. RAW264.7 cells were stimulated with LPS to simulate the inflammatory response in NS patients, and the regulation and mechanism of CASC15 on the inflammatory response were explored in this in vitro cell model. Serum CASC15 was upregulated in NS patients, and it had the ability to distinguish NS patients from NP patients. LPS stimulation increased the expression of CASC15 and simultaneously stimulated the secretion of inflammatory cytokines, while the knockdown of CASC15 alleviated the inflammatory response induced by LPS stimulation. Besides, serum miR-144-3p was reduced in NS patients, and luciferase reporter genes showed that miR-144-3p was a direct target of CASC15. Overexpression of CASC15 may promote the inflammatory response of NS by targeted regulating the expression of miR-144-3p, which may provide us with new insights in the treatment of NS.

## Introduction

Sepsis is a kind of systemic inflammation caused by the imbalance of the host’s immune response to infection [1]. Neonatal sepsis (NS) is a systemic infection occurring within 28 days of birth, which is a leading cause of neonatal death [1,2]. In 2002, the International Pediatric Sepsis Consensus Conference proposed that the specific definition of sepsis in children has significant differences in adult clinical signs and laboratory biomarkers [3]. NS differs from adult sepsis in epidemiology, pathophysiology, and even clinical management [4]. At present, most clinical criteria for NS are based on the combination of clinical signs and laboratory examination results [5,6]. Therefore, when the onset of NS occurs, it is of great significance for delaying the development of NS to make correct judgments and targeted interventions.

Long non-coding RNA (lncRNA) is a class of RNA molecules with a length of more than 200 nucleotides [7]. It has many functions and participates in cellular processes [8]. With the deepening of gene research, lncRNA has been found to play a key role in many physiological processes and diseases. Several lncRNAs are differentially expressed in the blood of healthy people and sepsis patients and are correlated with the severity of sepsis, indicating that lncRNAs may have the potential to be a diagnostic biomarker for sepsis [9]. Geng et al. revealed that lncRNA metastasis-associated lung adenocarcinoma transcript 1 (MALAT1) was expressed at elevated levels in the serum of patients with sepsis, and this gene could be used as a potential biomarker for the diagnosis of sepsis [10]. Wang et al. found that small nucleolar RNA host gene 16 (SNHG16) was down-regulated in NS through microarray analysis, and mechanistic experiments confirmed that SNHG16 could act as a ceRNA to regulate miR-15a/16 clusters, thus influencing inflammatory pathways [11]. CASC15 is a widely studied carcinogenic lncRNA, which is abnormally expressed to varying degrees in ovarian cancer, bladder cancer, lung cancer, and esophageal cancer [12,13]. In recent years, studies have found that CASC15 is abnormally elevated in diabetes mellitus complicated with chronic renal failure. Aude et al. found that CASC15 (also known as TLINC) was expressed in intrahepatic cholangiocarcinoma, and it was associated markedly with inflammatory response [14]. At present, there are relatively few related studies on CASC15 and sepsis, while most of the studies on CASC15 focus on inflammation.

Based on the above literatures, in the present study, we speculate that CASC15 may be abnormally expressed in sepsis and play a role in the development of inflammation. Therefore, the expression of serum CASC15 in NS patients and the clinical diagnostic value of CASC15 for NS were evaluated. Subsequently, we studied the role and possible mechanism of CASC15 in inflammatory response by constructing septic inflammatory cell model.

## Materials and methods

### Subjects and samples

Seventy-nine neonatal pneumonia patients and 80 neonatal sepsis patients were included in this study. The diagnostic criteria for NS is based on the criteria established in Kunming Conference on the Definition of Neonatal Sepsis in 2003, which mainly depends on clinical manifestations and blood pathogen detection results [15,16]. Newborns without signs or symptoms of sepsis who were diagnosed with pneumonia during routine consultations or vaccination in neonatal clinic were presented as the control group for the study. Premature infants, newborns with congenital malformation or chromosomal abnormalities were excluded from the study. Blood samples of all subjects were collected for laboratory examination before enrollment, and clinical indicators of the subjects were recorded and collected, which are summarized in Table 1. This study was implemented in the Neonatal Intensive Care Unit (NICU) after being permitted by the Ethics Committee of Shandong Provincial Hospital Affiliated to Shandong First Medical University (Ethical approval number: 2020–031). All the guardians of the newborns have signed written informed consent.

**Table 1.** Clinical data of the study population

Indicators	Pneumonia patients	Sepsis patients	*P*
	(n = 79)	(n = 80)	
Gender (male/female)	40/39	42/38	0.814
Body weight (kg)	3.51 ± 0.48	3.40 ± 0.48	1.21
Age (days)	11.13 ± 4.33	11.01 ± 5.32	0.882
IL-6 (pg/mL)	292.31 ± 50.83	368.50 ± 53.28	<0.001
IL-8 (pg/mL)	412.19 ± 41.17	454.08 ± 71.73	<0.001
TNF-α (pg/mL)	308.13 ± 40.60	374.91 ± 63.43	<0.001-
WBC (×10^9^/L)	14.93 ± 6.85	18.30 ± 6.25	0.001
CRP (mg/L)	11.25 ± 4.55	16.36 ± 5.39	<0.001
PCT (ng/mL)	1.56 ± 0.88	3.73 ± 1.39	<0.001

WBC, white blood cell; CRP, C-reactive protein; PCT, procalcitonin; IL-6, interleuk-6; IL-8, interleuk-8; TNF-α, tumor necrosis factor -alpha.

### Cell culture and cell transfection

The RAW264.7 cells were obtained from Cell Bank of the Chinese Academy of Sciences. Cells were cultivated in RPMI 1640 supplemented with 10% FBS and 1% Penicillin/streptomycin, and grown in an incubator containing 5% CO_2_ at 37°C. RAW264.7 cells were stimulated with LPS for a certain period of time, and an in vitro cell model of sepsis was obtained according to the previously published methods [17].

si-CASC15, si-NC, miR-144-3p mimic/inhibitor, mimic/inhibitor-NC were designed and synthesized by GenePharma company. Cell transfection was performed after the confluence of the cells was ~80%. In short, cells were seeded into 6-well plate, and then the above genes were transfected into cells by Lipofectamine 2000 following the manufacturer’s instructions, respectively.

### RNA extraction and real-time quantitative PCR (RT-qPCR)

5 ml of venous blood was taken from each participant and centrifuged at 1200 rpm for 5 min to obtain serum samples. Total RNA was extracted using TRIzol reagent in line with the manufacturer’s instructions for subsequent analysis. And then, RNA was reverse transcribed into cDNA using SuperScript II Reverse Transcriptase kit and PrimeScript™ RT reagent Kit, respectively. According to the methods and procedures provided in the instructions, the amplification was performed by an SYBR Green QuantiTect RTPCR Kit on Applied Biosystems 7900 Real-Time PCR System.GAPDH and U6 are stipulated to normalize CASC15 and miR-144-3p, respectively. The relative expressions of CASC15 and miR-144-3p were calculated by the 2^−ΔΔCt^ method.

### Enzyme-linked immunosorbent assay (ELISA)

The expression levels of various cytokines, such as IL-6, IL-8 and TNF-α, in the supernatant of RAW264.7 cells were quantitatively determined by ELISA [18]. ELISA was performed by IL-6 ELISA kit, IL-8 ELISA kit and TNF-α ELISA kit in accordance with description of products. The OD value at 450 nm was measured by a microplate reader.

### Dual-luciferase reporter assay

StarBase V2.0 predicted the binding sequence of CASC15 and miR-144-3p. To confirm the relationship between CASC15 and miR-144-3p, the dual-luciferase reporter assay was used to verify the relationship according to the previous published studies [19]. The 3-’UTR fragment of CASC15 containing the miR-144-3p binding site was cloned into the luciferase reporter vector to construct the wild-type (WT) and mutant (MUT) reporter vectors. The above reporter vectors were co-transfected with miR-144-3p mimic/inhibitor, mimic/inhibitor-NC into RAW264.7 cells using Lipofectamine 2000. The luciferase activity in the different groups was measured by a dual-luciferase reporter assay system.

### Statistical analysis

Data analyses were performed using SPSS 18.0 and GraphPad Prism 5.0. Data are shown as mean ± standard deviation (SD). *P* < 0.05 was known as statistically significant. Differences were assessed by Student t test, chi-square test and one-way ANOVA. ROC curve was structured to evaluate the diagnostic value of CASC15 regarding NS. Pearson correlation coefficient was used to estimate the correlation between CASC15 and clinical parameters. All tests were performed using at least three parallel experiments.

## Results

### Clinicopathological characteristics

A total of 159 individuals were recruited in this study, including 79 neonatal pneumonia patients and 80 neonatal sepsis patients. The clinicopathological characteristics of all subjects were summarized in Table 1. There were significant differences between NS patients and NP patients in terms of IL-6, IL-8, TNF-α, WBC, CRP, and PCT (all *P* < 0.05), while gender, age, and body weight were not statistically significant between the two groups (all *P* > 0.05).

### Serum CASC15 was elevated in NS group and had potential as a diagnostic biomarker

**Figure 1.**
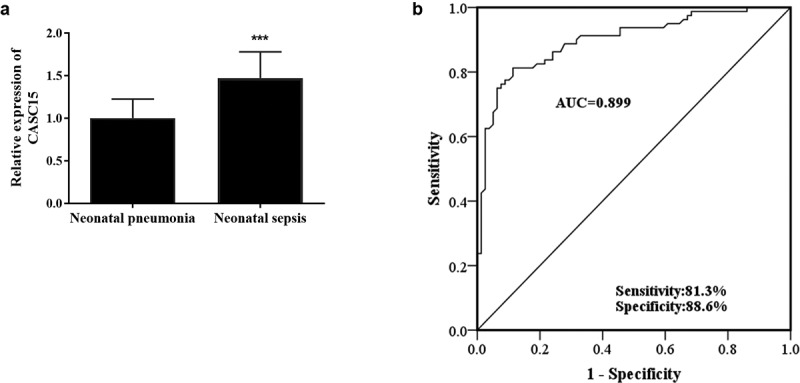
(a) Serum CASC15 was upregulated in neonatal sepsis patients. (b) ROC curve showed high diagnostic value of CASC15 in NS. ***P < 0.001 vs. neonatal pneumonia patients

To study the role of CASC15 in NS, RT-qPCR was used to detect CASC15 level in serum of all patients. Compared with the NP group, the relative expression level of serum CASC15 in NS patients was upregulated (Figure 1a, *p* < 0.001). Given the apparent dysregulation of serum CASC15 in NS patients, this study evaluated the clinical significance of serum CASC15 in the diagnosis of NS. In Figure 1b, the AUC of CASC15 level was 0.899, the sensitivity was 81.3%, and the specificity was 88.6%, indicating that serum CASC15 is capable of distinguishing NS patients from NP patients.

### Serum CASC15 was positively correlated with clinical indicators in NS patients

Pearson correlation coefficient was used to evaluate the correlation between serum CASC15 levels and clinicopathological indicators in NS patients. As presented in Table 2, it could be observed that serum CASC15 level in NS patients presents a significant positive correlation with the patients’ relevant clinical indicators, such as WBC, CRP, PCT, IL-6, IL-8, and TNF-α (all *P*< 0.01), showing that the level of CASC15 was correlated with the severity of NS.

**Table 2.** Correlation between lncRNA CASC15 and clinical characteristics

Characteristics	Correlation with lncRNA CASC15 (r)	*P-value*
WBC (×10^9^/L)	0.383	<0.001
CRP (mg/L)	0.451	<0.001
PCT (ng/mL)	0.465	<0.001
IL-6 (pg/mL)	0.439	<0.001
IL-8 (pg/mL)	0.367	0.001
TNF-α (pg/mL)	0.295	0.008

WBC, white blood cell; CRP, C-reactive protein; PCT, procalcitonin; IL-6, interleuk-6; IL-8, interleuk-8; TNF-α, tumor necrosis factor -alpha.

### CASC15 knockdown inhibited LPS-induced inflammatory response in RAW264.7 cells

**Figure 2.**
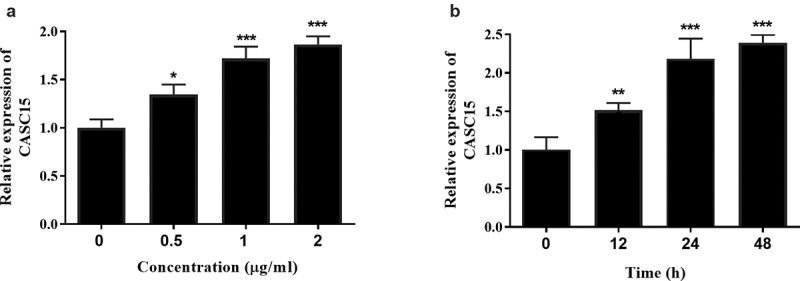
(a) The level of CASC15 enhanced with the increase of LPS concentration at the incubation time of 12 h. (b) The level of CASC15 enhanced with the increase of incubation time at the LPS concentration of 1 μg/ml. ***P < 0.001 and **P < 0.01

**Figure 3.**
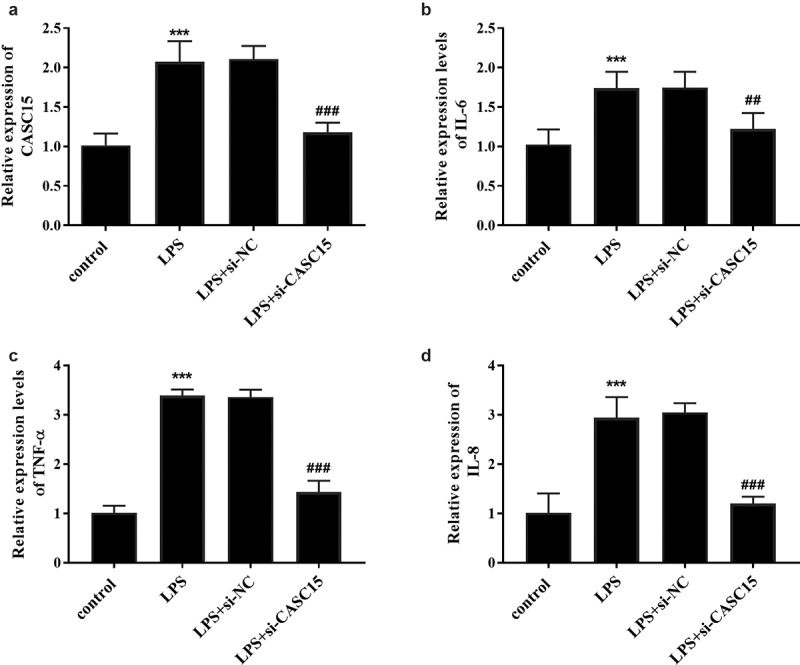
(a) Transfection of si-CASC15 reversed the LPS-induced elevation of CASC15 in RAW264.7 cells. The levels of (b) IL-6, (c) TNF-α, and (d) IL-8 were attenuated after transfection with si-CASC15. ***P < 0.001 vs. control group, ^###^*P*< 0.001, ^##^*P*< 0.01 vs. LPS group

LPS is a pivotal mediator of inflammatory response, which stimulates macrophages to release cytokines that promote inflammatory response, such as TNF-α, IL-8, and IL-6 [20]. Intermittent elevation of TNF-α and IL-1β in inflammatory response and sepsis has been reported [21]. To confirm the expression of serum CASC15 in an in vitro inflammatory cell model, we stimulated RAW264.7 cells with LPS and detected the gene expression of the cells. When incubation time was 12 h, the expression level of CASC15 increased in a concentration-dependent manner (Figure 2a, *p* < 0.05), while the expression level of CASC15 increased in a time-dependent manner at the LPS concentration was 1 μg/mL (Figure 2b, *p* < 0.01). Based on this result, we determined that the stimulating concentration of LPS to RAW264.7 cells in this study was 1 μg/mL and the incubation time was 24 h. In addition, we evaluated the role of CASC15 in LPS-induced inflammation by regulating intracellular CASC15 levels through in vitro transfection. The results showed that transfection of si-CASC15 significantly down-regulated the increase of CASC15 induced by LPS stimulation (Figure 3a, *p* < 0.001). As for cytokines, LPS-stimulation induced the increase of IL-6, TNF-α and IL-8 secreted by RAW264.7 cells, while down-regulation of CASC15 significantly reversed this phenomenon (Figure 3b-d, *P* < 0.01).

### CASC15 was directly regulated by miR-144-3p

**Figure 4.**
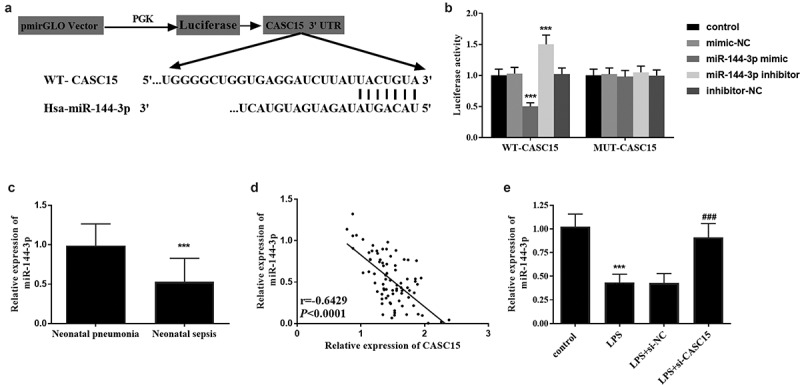
(a) Complementary sequences of CASC15 and miR-144-3p. (b) Luciferase reporter gene assay verified the relationship between CASC15 and miR-144-3p. (c) Serum miR-144-3p was downregulated in neonatal sepsis patients. (d) The level of miR-144-3p was negatively correlated with CASC15. (e) Transfection of si-CASC15 reversed the LPS-induced diminution of miR-144-3p in RAW264.7 cells. ***P < 0.001 vs. control group, ^###^*P*< 0.001 vs. LPS group

To study the mechanism of CASC15 in NS, we predicted that miR-144-3p and CASC15 had binding sites through StarBase v2.0 and the sequences were shown in Figure 4a. Subsequently, the results of luciferase reporter gene assay showed that luciferase activity in WT group attenuated after transfection with miR-144-3p mimic, while luciferase activity enhanced after transfection with miR-144-3p inhibitor, which was not observed in MUT group (Figure 4b, *p* < 0.001). At the same time, serum samples from all patients were analyzed and found that the level of miR-144-3p in NS patients was significantly lower than that in NP patients (Figure 4c, *p* < 0.001). Pearson correlation coefficient showed that the level of miR-144-3p decreased with the increase of CASC15 level (Figure 4d, *p* < 0.001). Meanwhile, Figure 4e showed that the level of miR-144-3p in cells decreased after LPS stimulation, and a marked upward trend in miR-144-3p level was observed after CASC15 knockout (*P* < 0.001). In general, the above results all proved the relationship between CASC15 and miR-144-3p.

## Discussion

NS is the important cause of neonatal disability death. Despite the significant improvement of neonatal intensive care technology and anti-infective treatment, the incidence and mortality of NS are still high. The culture of positive microorganisms in blood is often considered as the ‘gold standard’ for the diagnosis of bacterial NS. However, it does not seem to be the most reasonable test due to the long diagnostic cycle, which usually takes 2–3 days, it is prone to false-positive or false-negative results [22]. Early diagnosis is difficult, which is prone to misdiagnosis and missed diagnosis [23]. Therefore, it is of great clinical significance to search for sensitive and specific biomarkers as early warning indicators for the early diagnosis and treatment of NS.

The desired diagnostic biomarker ought to be easy to get and fast detection, whereas high sensitivity and specificity should also be the foremost essential characteristics of biomarkers. LncRNA and miRNA are widely displayed in blood, urine, saliva, and other body fluids, and its high stability in circulation makes it have the potential as a clinical reference biomarker [24]. In the past decades, many reports have pointed out the potential mechanism of lncRNA in sepsis. As reported previously, Bu et al. analyzed 28 up-regulated lncRNA and 61 down-regulated lncRNA in the circulation of NS patients by microarray technology [25], indicating that lncRNA was participated in NS. It was reported by Chen et al. that inhibition of lncRNA HOTAIR expression in mice with sepsis simultaneously downregulated TNF-α level and reduced inflammatory response in mice [26]. In the study, it was observed that the expression of serum CASC15 was increased in NS patients, and CASC15 exhibited good clinical diagnostic value for NS. In addition, the CASC15 expression showed a strong positive correlation with the inflammatory factors and other indicators of NS patients. All these results indicated that CASC15 played a role in NS and was positively correlated with the severity of NS.

Inflammation is one of the most important and basic clinical manifestations of sepsis [27]. RAW264.7 mouse macrophage line has been widely used to establish in vitro inflammation model [28]. RAW264.7 cells were stimulated with LPS to simulate the in vivo inflammatory status during sepsis in our study. It was observed that the expression level of CASC15 in RAW264.7 cells was enhanced after LPS stimulation. And we also found that the production of IL-6, TNF-α and IL-8 in cell models was significantly increased under LPS stimulation. Meanwhile, it was noteworthy that the adverse effects on cell model caused by LPS stimulation could be suppressed by the downregulation of CASC15. As mentioned above, after CASC15 (TLINC) overexpression, some inflammatory factor-related genes, such as IL-6, IL-8 and CXCL1, were induced to overexpress, leading to the formation of inflammatory response [14]. Taken together, downregulation of CASC15 can alleviate the inflammatory response caused by LPS induction. miR-144-3p, located in human chromosome 17q11.2 [29], has been reported to be associated with human myocardial infarction [30]. Multiple studies have shown that miRNAs play a role in sepsis and sepsis-induced heart damage [31]. In this study, we confirmed that CASC15 directly targeted miR-144-3p and negatively regulated the expression level of miR-144-3p. Besides, we also noticed that a decrease in the expression of miR-144-3p in the serum of NS patients. Many studies have confirmed our results at this stage. For example, Wei et al. reported that miR-144-3p was diminished in LPS induced sepsis cell model [32]. A study by Qin et al. showed that the expression of miR-144-3p in sepsis patients was significantly lower than that in healthy people through RNA sequencing and bioinformatics analysis [33]. Based on the above results, we speculated that in the sepsis cell model, overexpression of CASC15 may play a role in regulating inflammatory response by targeting miR-144-3p, thus promoting the development of sepsis. Nevertheless, there are still some limitations which should not be ignored in our study. In the current study, we did not further analyze the diagnostic value of CASC15 in sepsis, but preliminarily verified its ability to distinguish sepsis from pneumonia. In addition, we have not discussed the significance of CASC15 in the clinical outcome of septic neonates. The relationship between CASC15 and miR-144-3p, as well as how they regulate the inflammatory response, and even the influence of miR-144-3p on the inflammatory response, all need to be further discussed. It should be emphasized that our current study and understanding of lncRNA has just begun, and more studies on its functional mechanism and regulation are still needed to further apply lncRNA to the diagnosis and treatment of diseases.

The lncRNA and miRNA belong to non-coding RNA [34]. In recent years, increasing studies have shown that non-coding RNA is an important molecule involved in the regulation mechanism of sepsis, and the potential of non-coding RNA as a disease biomarker is being developed [35]. LncRNA such as NEAT1, HOTAIR, and MALAT1 have been identified as being significantly associated with the development of sepsis [36]. Bernadett et al. found that miRNA changes were significantly related to platelet activation in sepsis patients [37]. Jeffery et al. evaluated the role of non-coding RNA in the pathogenesis of sepsis and as a biomarker or treatment in a systematic study [38]. They found that although non-coding RNA seems to be a good biomarker and candidate drug for the treatment of sepsis, their differential expression in tissues complicates this process. Many studies have shown that circulating noncoding RNA has potential as a laboratory biomarker of sepsis, and it may be useful to further study the organ-specific transmission of these regulatory molecules.

## Conclusion

In conclusion, this study revealed that serum CASC15 is highly expressed in NS patients and verified that CASC15 has the potential to be a new diagnostic biomarker for NS. Besides, overexpression of CASC15 significantly promoted the production of IL-6 and other inflammatory factors in LPS-induced RAW264.7 cells, which may be an effect achieved through the targeted regulation of miR-144-3p level.

## Highlights

1. The expression level of CASC15 was increased in neonatal sepsis patient group, while miR-144-3p expression was decreased.

2. The highly expressed CASC15 is capable of distinguishing neonatal sepsis from neonatal pneumonia.

3. CASC15 was upregulated in LPS-induced RAW264.7 cells, and inhibition of CASC15 suppressed the production of inflammatory factors.

4. miR-144-3p has complementary sequence with CASC15, and its expression is negatively regulated by CASC15.
